# Sacral Microvascular Response in Older Adults Following Mechanical Loading Assessed by Photoplethysmography and Infrared Thermometry

**DOI:** 10.3390/ijerph22111759

**Published:** 2025-11-20

**Authors:** Catalina Jimenez Cerquera, Luz Edith Garzon, Alexandra María Patarroyo, Iván David Bañol, Rosa Nury Zambrano Bermeo

**Affiliations:** 1 Doctoral Program in Applied Sciences, Faculty of Basic Sciences, Universidad Santiago de Cali, Cali 760036, Colombia; catalina.jimenez00@usc.edu.co; 2 Nursing Program, Faculty of Health, Universidad Santiago de Cali, Cali 760036, Colombia; luz.garzon01@usc.edu.co (L.E.G.); alexandra.patarroyo00@usc.edu.co (A.M.P.); ivan.banol00@usc.edu.co (I.D.B.); 3 Programa de Enfermería, Facultad de Salud, University of Santiago de Cali, Cali 760036, Colombia

**Keywords:** temperature, aging, blood circulation, sacrum, pressure ulcers

## Abstract

Pressure injuries in institutionalized older adults with reduced mobility represent a relevant clinical problem due to their impact on quality of life and health costs. **Purpose:** This study aimed to describe the behavior of blood flow and tissue temperature in the sacral region and the significance of interindividual conditions in the post-load recovery of tissue in the context of pressure injuries. **Methods:** An observational study was conducted in 55 older adults living in a geriatric institution. Photoplethysmography and temperature signals were recorded at four times: at baseline (preload) and 15 s, 5 min, and 10 min post-load. The perfusion index was calculated, and two dichotomous variables were defined, Return b (flow) and Return t (temperature), to evaluate the physiological capacity to return to baseline conditions. Associations with clinical, functional, and diagnostic variables were explored. **Results:** The perfusion index showed usefulness as an indicator of microvascular recovery, but with variability between individuals. Return b was significantly associated with BMI and diagnosis of cardiovascular disease, while t-return was associated with body temperature and basal perfusion index. No significant associations were observed with sex, age or functional scales, state of consciousness, or risk of pressure injury. **Conclusions:** The study shows heterogeneous physiological responses to external load influenced by individual characteristics. Flow and temperature monitoring allows a functional approximation of injury risk, although more complex models are required to understand the nonlinear dynamics of tissue responses.

## 1. Introduction

Pressure injuries (PPL) are a public health problem with great clinical, economic, and ethical implications, especially in vulnerable populations such as institutionalized older adults [1,2]. These lesions, classified within the group of Dependence-Related Skin Lesions (CSDR), originate from the interaction between extrinsic factors and some other intrinsic conditions of the individual [3,4,5], such as reduced mobility, malnutrition, advanced age, and perfusion alterations [5]. Authors such as García Fernández describe multifactorial models in relation to the development of pressure injuries and express the complexity of the phenomenon [5]. Various studies have shown that pressure injuries can be prevented [6,7,8,9]; however, the most widely used risk assessment scales, such as the Braden [10] and Norton scales [11], have limitations related to subjectivity in application and experience on the part of the evaluator [12].

However, beyond individual errors, the occurrence of these adverse effects is also linked to failures in organizational aspects, working methods, or tools. In this sense, the assessment of care needs by health personnel is the first step to detect risk situations that can lead to adverse events. To carry this out, a wide variety of instruments are routinely used that can vary depending on the clinical context, units, and hospitals [8,9]. The use of these instruments is essential to identify real or potential problems in patients, although it increases the administrative burden and limits the time of direct care [13]. Direct care, on the other hand, is associated with a decrease in mortality, a higher quality of care, greater user satisfaction, an improvement in the functional capacity of patients, and a reduction in pressure injuries and falls [13]. In addition, understanding not only who is at risk, but also when they are most at risk, would allow for prioritizing care, optimizing resources, considering these factors in staffing models, and improving patient comfort. For example, if a nurse has five patients and four of them are at risk based on the standard bedside risk assessment, but only one is at risk based on the time of risk, the nurse will be able to prioritize care more efficiently, directing interventions to those who need them most [14].

In the hospital setting, particularly in the prevention of injuries due to reduced mobility, a previous study revealed that nursing staff face difficulties in managing the care of patients with reduced mobility. Although healthcare professionals acknowledge the importance of providing high-quality care, their efforts are often constrained by the multiple priorities and demands inherent to clinical practice. Addressing the needs of patients with reduced mobility or functional dependence requires sufficient time, qualified human resources, and the support of appropriate technology [15,16,17].

This situation has prompted the search for more objective strategies, which incorporate quantifiable physiological measurements in real time [18]. Thus, blood flow and skin temperature have been postulated as complementary tools for the assessment of tissue involvement [19,20]. Reactive hyperemia, understood as a transient increase in flow after a period of ischemia [8], could constitute a preliminary marker of subclinical tissue damage [21,22]. In fact, it has been shown that certain changes in these responses can be associated with damage to the underlying muscle tissue, even before it can be evidenced in the subcutaneous tissue as a visible injury [23].

Several investigations have shown that alterations in perfusion and skin temperature act as early physiological markers of tissue response under mechanical load, even before visible damage becomes evident [7,8,18,19,20,22]. Photoplethysmography (PPG) has emerged as a non-invasive optical technique capable of monitoring microvascular behavior and detecting changes related to pressure-induced stress on soft tissues. Complementarily, skin temperature measurements offer additional information that may be associated with vascular compromise. The combined use of these physiological signals provides a more objective framework for evaluating tissue behavior following mechanical loading and may help overcome the limitations of conventional risk assessment tools, which largely rely on the subjectivity of the clinician applying them [12,13,14].

However, clinical evidence in older adults at risk remains limited, and further studies are needed to understand how these physiological responses manifest according to individual characteristics. In this context, the present study aimed to describe the behavior of blood flow and sacral temperature, as well as the role of interindividual factors, in the post-load recovery response of institutionalized older adults at risk of pressure injury.

## 2. Materials and Methods

### 2.1. Study Design

A prospective, descriptive, exploratory, and observational study was developed in a cohort of institutionalized older adults with the aim of characterizing the behavior of blood flow and sacral temperature of older adults in the face of sustained pressure exposure, considering variables such as age, nutritional status, and reported diagnoses. The exploratory scope of this manuscript corresponds to an early phase of a larger project and focuses on descriptive and bivariate findings without the inclusion of multivariate or predictive models.

This study is derived from the macro-project entitled “Relationship of blood flow and temperature as a function of time with reactive hyperemia as an early indicator of pressure injuries”, approved by the Ethics Committee of the Faculty of Health of the University of Santiago de Cali. All procedures were carried out in accordance with the ethical principles established in the Declaration of Helsinki [24] and protected by Resolution 8430, which establishes scientific, technical, and administrative standards for health research in Colombia [25].

### 2.2. Participants

The target population consisted of institutionalized older adults in a geriatric hospital in Cali, Colombia. A total of 55 participants were included based on the following criteria: hospital stay > 48 h, intact sacral region with no previous or recent injuries, age ≥ 60 years, and moderate to severe functional dependence (Barthel Index < 40) [26]. Participants with active dermatological lesions or evidence of fibrous scar tissue in the evaluation area, lesions associated with moisture or medical adhesives, presence of visible skin erythema in the sacral area at admission, and patients who suffered from skin diseases of any other origin or with a history of pressure ulcers in the evaluated area were excluded.

### 2.3. Measurement Protocol

The protocol consisted of three uninterrupted phases and four measurement time points, performed under real clinical conditions without modifying the patient’s usual care environment. Mechanical load was not standardized as it depended on each patient’s body weight and the type of support surface; this decision was intentional in order to preserve ecological validity.

Phase 1—Preload. Baseline Establishment: The patient was positioned in lateral decubitus, and an initial measurement was made using a non-invasive photoplethysmography (PPG) prototype that emits red and infrared LED light. This measurement served as a baseline of perfusion and sacral temperature.

Phase 2—Loading: The patient remained supine for 10 min. During this phase, no measurements were made, allowing the continuous application of pressure on the sacral region. Continuous monitoring was not implemented due to patient tolerance and workflow constraints.

Phase 3—Afterload: After the repositioning of the patient to lateral decubitus, 3 measurements were made to capture the physiological response. The first measurement was made at 15 s post-turn, the second measurement at 5 min post-turn, and the third measurement at 10 min post-turn.

In total, four blood flow and temperature measurements were obtained per participant, allowing the evolution of the behaviors of the variables after exposure to mechanical load to be observed.

The measurement protocol was structured considering the temporal dynamics previously described for reactive hyperemia and microvascular responses in tissues subjected to mechanical pressure. Previous studies, such as that by Bergstrand et al. (2014), established 10 min loading and post-loading periods based on evidence indicating that reactive hyperemia typically lasts for a duration comparable to that of the ischemic stimulus [8].

Accordingly, the present study defined specific measurement windows for both the loading and post-loading phases, including baseline (t_0_), 15 s post-unloading (t_1_), 5 min post-unloading (t_2_), and 10 min post-unloading (t_3_). This design aimed to capture the early, intermediate, and late phases of the microvascular and thermal responses, enabling the characterization of both the peak response and the potential recovery or return toward baseline [7].

### 2.4. Collection of Clinical and Anthropometric Data

At the same time, clinical and contextual data were collected from each participant from institutional records and standardized clinical assessments. Variables included

- Age, sex, and body temperature;
- Relevant medical diagnoses reported;
- Body mass index (BMI) and nutritional status [27];
- Weight and height (data were estimated based on anthropometric measurements of patients) [28];
- Braden [10] and Barthel [26] risk assessment scales and the Glasgow [29] scale.

These variables were used to describe the interindividual profiles of the participants and explore their possible relationship with the physiological responses of flow and skin temperature observed. Prior to data collection, internal functional tests were performed to ensure signal stability and repeatability, considering the exploratory nature of the study.

### 2.5. Photoplethysmography and Infrared Temperature Device

A non-invasive system was used to measure blood flow through photoplethysmography (PPG), and skin temperature was measured using an infrared sensor. The PPG module included red and infrared LEDs with central emission wavelengths of 660 nm and 880 nm, respectively [7,8], wavelengths commonly used to detect microvascular blood volume variations in superficial tissues.

The device underwent internal functional tests to verify signal stability and repeatability prior to data collection, considering the exploratory nature of the study. Although detailed specifications are not disclosed for technical confidentiality reasons, the system was experimentally benchmarked against a reference laser Doppler flowmetry device and comparatively validated using Doppler ultrasound, demonstrating comparable performance to standard methods and adequate discriminatory capacity.

To minimize signal noise during acquisition, the sacral region was carefully inspected to ensure that the skin was clean, dry, and free of visible lesions or scars, in accordance with previous protocols for optical measurements in soft tissues (Bergstrand et al., 2014) [8]. In addition, potential electronic interference sources were avoided, and digital filters were applied during signal processing to reduce artifacts and improve measurement stability [7].

Photoplethysmography was selected for its sensitivity to pressure-induced microvascular changes, as supported by previous evidence, while infrared temperature measurement provided complementary information on skin thermal behavior and its relationship with local perfusion. Mean values were calculated for each measurement zone.

### 2.6. Signal Processing and Variable Extraction

The PPG signal was analyzed by separating its two components: a continuous (DC) component, representing the constant absorption of light by tissue, and a pulsatile (AC) component, reflecting rhythmic variations associated with cardiac cycles or activity. From these components, the perfusion index (PI) was derived using the standard formula PI = AC/DC [8].

Temperature data were incorporated as a contextual variable, providing complementary information on the thermal behavior of the tissue and its relationship with perfusion dynamics, linked to inflammatory, vasodilatory, and ischemic processes.

It is important to note that the raw PPG signal is expressed in arbitrary units (a.u.), as is standard in optical microcirculation techniques such as laser Doppler flowmetry. These measurements do not represent absolute perfusion values but rather relative and time-dependent fluctuations, allowing analysis of trends and microvascular recovery potential for each participant.

### 2.7. Data Analysis

Descriptive statistical analyses were applied to characterize the distribution and central tendencies of the collected data. For data processing, they were grouped according to the corresponding variables and coded, converting the qualitative responses into numerical data. In addition, outliers were reviewed, and for missing data, an imputation strategy was applied using the KNN (k-Nearest Neighbors) algorithm [30]. The data was captured and recorded in an Excel table and processed using a Python 3.10 script run in Google Colaboratory (2024 version) [31]. The quantitative variables were described by measures of central tendency (mean and median) and dispersion (standard deviation) [32], while the qualitative variables were described by absolute and relative frequencies [33].

The Barthel Index was analyzed in two ways: (1) as a continuous quantitative variable, using the total score for descriptive and bivariate analyses, and (2) as a categorical variable, applying clinical cut-off points to classify functional dependence (total, severe, and moderate). This dual approach preserved the metric information of the score while enhancing clinical interpretability.

In addition to the descriptive analysis, bivariate analyses [34] were performed to explore differences in numerical and categorical quantitative variables between groups defined by dichotomous categorical variables, including whether flow and temperature returned to baseline levels. To achieve this, the Shapiro–Wilk normality test was applied in each group [35], followed by the homogeneity of variances test (Levene) [36]. According to the statistical assumptions, parametric (Student’s T) [37] and non-parametric (Mann–Whitney U) tests [38] were used to compare the level of significance of the variables between the groups, as well as the Chi-square test [39] and Fisher’s exact test [40]. The analytical approach was limited to exploratory analyses and did not include complex multivariate modeling, which will be reported in subsequent studies derived from the macro-project.

## 3. Results

### 3.1. Exploratory and Univariate Analysis

Data were collected from 55 institutionalized older adults. A total of 85.5% of participants completed all measurement phases, providing valid records for the variables analyzed. On the other hand, 12.7% of patients have measurement records up to minute 5 of the post-load phase (t_2_); these interruptions were mainly associated with factors related to difficulties in tolerance to the procedure. In addition, only one patient had records of a single measurement in the preload (t_1_), associated with situations of irritability or low tolerance to manipulation due to neurological involvement, so it was necessary to impute the data through a KNN algorithm [30] to proceed with the analysis.

The sample consisted of 55 institutionalized older adults, with a balanced sex distribution and a high prevalence of underweight and multimorbidity. Table 1 summarizes the main sociodemographic and anthropometric characteristics of the participants.

The sample showed a high prevalence of multimorbidity, severe functional dependence, and elevated risk for pressure injury development. Table 2 summarizes the clinical characteristics and risk profile of the participants.

**Figure 1:**
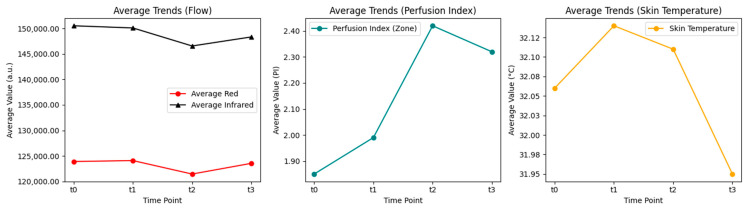
Average temporal trends of blood flow, perfusion index, and skin temperature after mechanical loading.

The average perfusion signal for the group showed temporal variations following mechanical loading. For the red channel, mean values were 123,904.27 a.u. at baseline (t_0_), 124,085.40 a.u. at 15 s (t_1_), 121,448.59 a.u. at 5 min (t_2_), and 123,547.62 a.u. at 10 min (t_3_). For the infrared channel, the mean baseline value was 150,539.40 a.u. (t_0_), followed by 150,112.96 a.u. (15 s or t_1_), 146,572.12 a.u. (5 min or t_2_), and 148,350.56 a.u. (10 min or t_3_). While both channels demonstrated post-pressure fluctuations, only the red channel approached its initial reference value by minute 10 (Figure 1).

The perfusion index (PI), derived from the ratio of the AC and DC components of the photoplethysmographic signal, showed a progressive rise following mechanical loading. Mean PI values were 1.85 at baseline (t_0_), 1.99 immediately after unloading (t_1_), 2.42 at minute 5 (t_2_), and 2.32 at minute 10 (t_3_). Although a mild decline occurred after the peak at minute 5, the signal did not return completely to its baseline level by the end of the observation period (Figure 1).

In terms of skin temperature, the following values were recorded at each time: 32.06 °C as the starting point (t_0_), 32.14 °C immediately following the load (t_1_), 32.11 °C five minutes after the load (t_2_), and 31.95 °C ten minutes after the load (t_3_). In keeping with the behavior anticipated in a mild reactive hyperemia response, this pattern showed a brief rise followed by a slow decline towards baseline (Figure 1).

There was a significant amount of variation in the participants’ ages. Given the high percentage of people who were classed as underweight, anthropometric markers including weight and body mass index (BMI) displayed a skewed distribution towards low values. As a reflection of the distinctive variability of institutionalized people, height also displayed high extremes. Within the anticipated physiological range, the core body temperature displayed a narrow distribution at about 36.6 °C.

The Glasgow score showed a distribution with a ceiling effect in comparison to the clinical scales, meaning that most subjects achieved the maximum score of 15 points. The Braden [10] and Barthel [26] scales, on the other hand, displayed more dispersion, indicating variations in the degree of functional dependency and the likelihood of pressure injuries. Table 2, which provides an overview of the individuals’ clinical features, displays these findings.

### 3.2. Bivariate Analysis: Comparison to Return to Baseline

The sample was split into return and non-return groups for the study of temperature and blood flow findings. This analysis was carried out based on how each variable behaved in relation to its baseline value using the criteria outlined in Section 2.

Based on the above, the bivariate analyses outlined below were developed to compare the quantitative and categorical variables between the groups defined by return or non-return to baseline in order to comprehend the relationship between tissue recovery and the clinical or physiological characteristics of the participants.

#### 3.2.1. Analysis of Quantitative Variables vs. Return to Baseline of Blood Flow

In the case of blood flow, the perfusion index (PI) was used as a robust indicator of tissue perfusion since it represents the relationship between the pulsatile (AC) and non-pulsatile (DC) components of the photoplethysmographic signal, attenuating the impact of noise or variations in the signal. Based on this indicator, the dichotomous variable was defined as Return b, and cases whose difference between the baseline value (t0) and the value at minute 10 (t3) was within the range of the baseline standard deviation were classified as return [32], which allows interindividual physiological variability to be incorporated into the analysis.

To explore the differences between the *return* and *no return* groups, the Shapiro–Wilk [35] normality test was applied to each numerical variable in both groups, and the Levene test [36] was used to assess the homogeneity of variances. Based on these results, the most appropriate statistical test was selected: Student’s *t* test was used for independent samples [37] when the assumptions of normality and homoscedasticity were met, and the Mann–Whitney U test was used [38] otherwise.

In the results, statistically significant differences were observed for the following variables:

- **Weight** (*p* = 0.0179) and **IMC** [27] (*p* = 0.0472): both with normal distribution and homogeneous variances, evaluated with Student’s T-test [37];
- **IP Zone 3** (*p* = 0.0181): with non-normal distribution in at least one of the groups, so the Mann–Whitney U test was used [38].

The rest of the variables did not present statistically significant differences. This analysis allowed us to identify variables that could be associated with a better or worse recovery capacity of tissue perfusion after exposure to mechanical load, valuable information for the design of future evaluation and preventive monitoring strategies.

#### 3.2.2. Analysis of Categorical Variables and Return to Baseline of Blood Flow

The possible association between the variable “Return to baseline” and various categorical variables was analyzed using the Chi-square test [39] or Fisher’s exact test [40] according to the assumptions of each test. The variables included in this analysis were clinical diagnoses coded as binary variables, classification of nutritional status, categories of clinical scales (Barthel [26], Braden [10], and Glasgow [29]), and the combined classification of systolic and diastolic blood pressure. When the contingency tables presented expected frequencies less than 5, Fisher’s exact test [40] was applied. In the other cases, Pearson’s Chi-square test [39] was used.

The results showed statistically significant associations between return to baseline and the following variables:

- **Nutritional status** (*p* = 0.0231), qualitative interpretation of BMI [27];
- **Diagnosis of cardiovascular disease** (*p* = 0.0119).

The remaining categorical variables did not show statistically significant associations; however, they should be explored in future studies with larger sample sizes. These findings suggest that cardiovascular comorbidity and nutritional status may influence the probability of physiological recovery after mechanical loading, highlighting their potential role as modulatory factors of cutaneous hemodynamic behavior.

#### 3.2.3. Analysis of Quantitative Variables vs. Return to Baseline of Temperature

Regarding the thermal response, the analysis of **T-Return** showed that a considerable number of patients did not return to their basal temperature 10 min after the shock. In particular, **core body temperature** showed a significant difference between groups (*p* = 0.0099), while other measurements such as object or ambient temperature did not present statistically relevant differences.

As in the flow analysis, no significant associations were observed between **Return T** and demographic variables such as age, sex, or BMI [27]. However, high inter-individual variability was identified, reinforcing the need for customized approaches to risk analysis.

#### 3.2.4. Analysis of Categorical Variables vs. Return to Baseline Temperature

The possible association between the return to basal temperature after mechanical discharge (t-return) and different categorical variables was explored using the Chi-square test [39] or Fisher’s exact test [40] according to the type of variable and the expected counts. No statistically significant associations were found between t-return and the categorical clinical variables included in the analysis. Nutritional status, sex, and classifications of the clinical scales (Barthel [26], Braden [10], and Glasgow [29]) showed no significant differences between the groups with and without thermal return.

Likewise, when evaluating binary clinical diagnoses (e.g., presence or absence of pathologies by systems), no statistically significant associations were observed. All the *p*-values obtained by testing for these variables were greater than 0.05, suggesting that the presence of diagnoses such as cardiovascular, respiratory, infectious, neurological, or metabolic pathologies was not related to the probability of returning to basal temperature after pressure.

Although the Barthel Index [26] showed a trend close to significance (*p* = 0.0545), it did not reach the conventional statistical threshold, so it is recommended to evaluate this possible pattern in studies with larger sample sizes.

## 4. Discussion

According to the results obtained, it was possible to characterize the behavior of blood flow and thermal responses in the sacral region of institutionalized older adults subjected to mechanical loading. An incomplete recovery of both blood flow and skin temperature was observed in a subgroup of participants during the 10 min observation window. This pattern may indicate an alteration in microvascular reactivity, consistent with the findings of Lupiáñez-Pérez et al. (2021) [42], who associated aging and comorbidities with decreased perfusion capacity and impaired vascular reactivity [43].

In this context, the differences observed between the red (660 nm) and infrared (880 nm) channels provide complementary insights into vascular dynamics. Infrared light, which penetrates deeper into the tissue, reflects more stable hemodynamic variations, whereas the red channel—being more sensitive to oxyhemoglobin changes—captures superficial fluctuations more immediately after loading and unloading. This dual behavior suggests that recovery processes involve both superficial and deeper microvascular mechanisms, consistent with the cutaneous physiology described in previous optical flow-assessment studies [8].

The perfusion index (PI), obtained from the ratio between the AC and DC components of the photoplethysmographic signal, proved to be a robust indicator of relative perfusion variations as it partially reduces the influence of transient artifacts. However, its interpretation should consider interindividual variability and the clinical context, since factors such as dry skin, edema, or even microangiopathies may alter the morphology of the signal. The dispersion observed in PI trajectories among participants aligns with the findings of Lupiáñez et al. (2021), who reported both regional and interindividual differences in tissue-flow recovery dynamics [42].

The rise in temperature immediately following pressure relief, followed by a gradual decline during the recovery period, is consistent with the observations of Källman et al. (2015), who associated these thermal changes with reactive hyperemia followed by compensatory vasoconstriction [7]. This thermal pattern, together with the variability in returning to baseline, may reflect alterations in vascular autoregulation or cutaneous blood-flow redistribution—phenomena frequently observed in older adults with multiple comorbidities [42].

Bivariate analysis revealed no statistically significant differences in physiological recovery—neither in blood flow (Return B) nor temperature (Return T)—with respect to sex, age, functional scales (Barthel, Braden, and Glasgow), or most of the grouped clinical diagnoses. This finding contrasts with previous studies that reported sex-related differences in vascular physiology and in PPG-derived signal characteristics, such as amplitude, stability, and reperfusion rate [44,45]. This suggests that, although such physiological differences may exist, they do not necessarily translate into a differential capacity for recovery after mechanical loading, at least not significantly within our sample. However, this study did not specifically include the analysis of the reperfusion index, which could be a more sensitive indicator of vascular restoration; thus, future studies should explore this aspect further, as several authors (Fellahi et al., 2014 [46]; Keller et al., 2023 [47]; Rasica et al., 2022 [48]; Citherlet et al., 2024 [49]) have reported slower recovery rates in women when analyzing this parameter.

In contrast, significant associations were identified between Return B and variables related to body composition—particularly weight, body mass index (BMI), and its qualitative interpretation (nutritional status)—as well as with the perfusion index (PI) measured in zone 3, corresponding to the final time point in the post-load phase. These findings support the hypothesis that individual physiological characteristics, especially those related to body composition and peripheral perfusion, play a determining role in the vascular response to pressure. In line with this, Citherlet et al. (2024) described lower reperfusion rates and slower microvascular responses in older adults, which could help explain part of the variability observed in the present study [49,50].

Although cardiovascular diagnosis was the only clinical condition that was significantly associated with b-return (*p* = 0.0119), suggesting that people with cardiovascular disease may have reduced reperfusion capacity, possibly due to arterial stiffness or endothelial dysfunction [51], this observation should be interpreted cautiously, as the present study did not directly assess vascular stiffness or endothelial function. Nevertheless, the pattern observed may be consistent with previous evidence describing reduced microvascular reactivity in individuals with chronic cardiovascular conditions [51].

Regarding the thermal response (Return T), significant associations were observed with baseline body temperature and the PI in zone 0 (baseline). These results indicate that both the initial thermal state and basal perfusion could influence the likelihood of temperature recovery after pressure removal. A limited thermal response may indicate alterations in vascular autoregulation or in the redistribution of cutaneous blood flow, mechanisms that are frequently compromised by aging and general health conditions.

The findings of this study provide valuable insight into the physiological mechanisms and tissue responses associated with the risk of pressure injuries in institutionalized older adults. The implementation of flow (PPG) and temperature sensors enabled the characterization of microvascular behavior following mechanical loading, complementing conventional clinical scales such as Braden [10] or Barthel [26], which alone do not dynamically capture perfusion-related data. The inclusion of these physiological variables could contribute to a more objective classification of patient risk.

Although a delayed or incomplete recovery of perfusion or temperature return to baseline may suggest reduced tissue resilience, this study did not include a longitudinal follow-up to determine whether participants subsequently developed pressure injuries [52]. Therefore, these findings should be interpreted as part of the broader understanding of microvascular and physiological reactivity rather than as predictive indicators of lesion development, as further research is required to examine complex interactions between interindividual patient conditions through non-parametric modeling approaches.

In line with this, future studies incorporating continuous monitoring during hospitalization could help determine whether these physiological patterns anticipate the onset of pressure injuries. Nonetheless, the operationalization of binary variables such as *Return B* and *Return T* represents a methodological contribution that may facilitate their integration into clinical decision-support systems for physiological monitoring and early detection in geriatric or long-term care contexts.

This study also presents several limitations that should be acknowledged. Although the sample size was sufficient to identify statistically significant differences in certain variables related to physiological recovery during the post-load phase, its small size and the recruitment of participants from a single institution limit both statistical power and generalizability. Because the objective was to capture responses under real clinical conditions, the mechanical load applied was not standardized, as it depended on each participant’s body weight and the type of support surface used. This approach preserved ecological validity but introduced variability that was difficult to control. Furthermore, since continuous recording was not performed during the loading phase, it was not possible to identify dynamic phenomena such as pressure-induced vasodilation.

In addition, since continuous recording was not performed during the loading phase, it was not possible to identify dynamic phenomena such as pressure-induced vasodilation. There were cases with missing values in the measurements, for which an imputation procedure was applied using the k-Nearest Neighbors algorithm; however, the use of this technique in small samples may not fully reflect biological variability, so the findings should be interpreted with caution. Furthermore, this manuscript presents only exploratory and descriptive results, consistent with the pedagogical purpose and the defined scope of this publication. Nonetheless, future studies derived from the same project will incorporate additional models to characterize more complex interactions between clinical and physiological variables.

## 5. Conclusions

This study demonstrated the usefulness of non-invasive monitoring of flow signals (PPG) and tissue temperature to characterize the microvascular response to external loads in institutionalized older adults. From the temporal analysis of the signals and the application of indicators such as the perfusion index (PI), differentiated patterns of physiological recovery were identified that were not always coincident between flow and temperature nor homogeneous between individuals.

The operationalization of dichotomous variables [53], such as Return b (flow) and Return t (temperature), allowed us to evaluate in a complementary way the capacity to return to baseline conditions after the removal of the load, revealing significant associations with variables such as nutritional status or BMI [27], body temperature, and the presence of cardiovascular pathologies.

Although no significant differences were observed according to sex or the functional scales used, these results highlight the role of individual physiological factors in tissue reperfusion, which is key to improving early detection of pressure injury risk. The need for studies with larger sample sizes and continuous follow-up during loading and post-loading is suggested, including additional variables such as institutional length of stay in order to strengthen the findings and move towards integrated predictive models. Although the analysis included inferential tests, multivariate or non-parametric models were not incorporated to identify more complex or nonlinear relationships.

## 6. Patents

The prototype described and employed for data acquisition in this study is under review for patent protection. The intellectual property application is currently in process and not yet publicly disclosed.

## Figures and Tables

**Table 1:** Sociodemographic and anthropometric characteristics of the participants.

Category	Variable	n	%
Comorbidities *	≥2 chronic comorbidities	48	87.30
	Neurological	41	74.55
	Cardiovascular	36	65.45
	Psychiatric	20	36.36
	Endocrine/metabolic	11	20.00
	Other comorbidities **	13	23.64
Functional status (Barthel)	Total dependence	23	41.82
	Severe dependence	22	40.00
	Moderate dependence	10	18.18
Consciousness (Glasgow)	Alert (15/15)	35	63.64
	Sleepy	14	25.45
	Lethargic	4	7.27
	Dazzled	2	3.64
Risk of pressure injury (Braden)	High risk	27	49.09
	Moderate risk	19	34.55
	Low risk	2	3.64
	No risk	7	12.73

* Comorbidity categories are non-exclusive, as participants could present more than one diagnosis. ** “Other comorbidities” include less frequent conditions such as respiratory, renal, musculoskeletal, dermatological, and infectious pathologies and other diagnoses.

**Table 2:** Clinical characteristics and risk profile of the participants.

Variable	Mean ± SD	Min	Max
Age (years)	81.62 ± 10.47	61	105
Weight (kg)	39.17 ± 10.48	17.61	58.12
Height (m)	1.52 ± 0.13	1.24	2.16
BMI (kg/m^2^)	16.83 ± 3.39	8.6	22.64
Category		n	%
Nutritional status (BMI WHO) [41]			
Low weight		35	63.64
Normal weight		20	36.36
Sex			
Male		28	50.91
Female		27	49.09

## Data Availability

The data presented in this study are available upon request from the corresponding author. The data are not publicly available due to privacy and ethical restrictions.

## Abbreviations

The following abbreviations are used in this manuscript:

LPP	Pressure injuries
PPG	Photoplethysmography
PI	Perfusion index
BMI	Body mass index
LCRD	Dependence-related skin lesions
KNN	K-nearest neighbors
WHO	World Health Organization
FC	Heart rate
T° CORP	Body temperature
AC	Alternating current component of the photoplethysmographic signal; represents the pulsatile (dynamic) variation associated with arterial blood flow
DC	Direct current component of the photoplethysmographic signal; represents the non-pulsatile (static) baseline related to tissue and venous blood absorption.
